# A Physiologically Based Pharmacokinetic Model for the Assessment of Infant Exposure to Persistent Organic Pollutants in Epidemiologic Studies

**DOI:** 10.1289/ehp.0800047

**Published:** 2008-11-10

**Authors:** Marc-André Verner, Pierre Ayotte, Gina Muckle, Michel Charbonneau, Sami Haddad

**Affiliations:** 1 Département des sciences biologiques, Centre Toxen, Université du Québec à Montréal, Montréal, Québec, Canada;; 2 Centre de recherche du Centre Hospitalier Universitaire de Québec – Centre Hospitalier de l’Université Laval, Université Laval, Québec, Québec, Canada;; 3 Institut National de la Recherche Scientifique – Institut Armand-Frappier, Université du Québec, Laval, Québec, Canada

**Keywords:** epidemiology, exposure assessment, infants, persistent organic pollutants, physiologically based pharmacokinetic modeling

## Abstract

**Background:**

It has been suggested that pre- and postnatal exposure to persistent organic pollutants (POPs) can promote several adverse effects in children, such as altered neurodevelopment. Epidemiologic studies to date have relied on the analysis of biological samples drawn pre- or post-natally for exposure assessment, an approach that might not capture some key events in the toxicokinetics of POPs.

**Objectives:**

We aimed to build a generic physiologically based pharmacokinetic (PBPK) modeling framework for neutral POPs to assess infant toxicokinetic profiles and to validate the model using data on POP levels measured in mothers and infants from a Northern Québec Inuit population.

**Methods:**

The PBPK model developed herein was based upon a previously published model to which an infant submodel was added. Using the model and maternal blood levels at the time of delivery, exposure to 1,1-dichloro-2,2-bis(*p*-chlorophenyl)ethylene (*p,p*′-DDE), 1,1,1-trichloro-2,2-bis(*p*-chlorophenyl)ethane (*p,p*′-DDT), hexachlorobenzene (HCB), β-hexachlorocyclohexane (β-HCH), 2,2′,3,4,4′,5′-hexachlorobiphenyl (PCB-138), 2,2′,4,4′,5,5′-hexachlorobiphenyl (PCB-153), and 2,2′,3,4,4′,5,5′-heptachlorobiphenyl (PCB-180) in mothers was estimated to subsequently simulate infant blood, breast milk, and cord blood POP concentration. Simulations were then compared with corresponding measured levels through Spearman correlation analyses.

**Results:**

Predictions were highly correlated with measured concentrations for PCB-153, PCB-180, PCB-138, HCB, and *p,p*′-DDE (*r* = 0.83–0.96). Weaker correlations were observed for *p,p*′-DDT and β-HCH for which levels were near the limits of detection.

**Conclusion:**

This is the first study to validate a PBPK model of POPs in infants on an individual basis. This approach will reduce sampling efforts and enable the use of individualized POP toxicokinetic profiles in the epidemiologic studies of POP adverse effects on child development.

Many epidemiologic studies suggest that pre- and postnatal exposure to persistent organic pollutants (POPs) can promote several adverse effects in children, such as altered neurodevelopment (Eskenazi et al. 2008; Koopman-Esseboom et al. 1996; Ribas-Fito et al. 2001; Rosas and Eskenazi 2008; Walkowiak et al. 2001). Although environmental levels of most POPs declined after their use was restricted in many countries, these compounds are still found in most human tissues and blood samples. Moreover, some persistent chemicals, such as polybrominated diphenyl ethers (PBDEs) and 1,1,1-trichloro-2,2-bis(*p*-chlorophenyl)ethane (*p,p*′-DDT), are still produced and widely used.

Prenatal and early postnatal periods are critical for the developing brain. Mild endocrine disruption during these time windows can lead to deficits in neuropsychological functions in infants, and these effects are specific to the time when hormonal dysregulation occurs (Zoeller and Rovet 2004). Therefore, timing of exposure to endocrine-disrupting compounds during early stages of development is critical. Epidemiologic studies to date have relied on diverse biologic samples drawn prenatally, at birth, or postnatally to investigate relationships between exposure to POPs and various outcomes pertaining to health and development. Although multiple samples provide information on the overall exposure in infants, this approach is costly, time-consuming, and subject to ethical limitations and might not capture some key events in the lifetime toxicokinetic profiles of POPs. Therefore, new exposure assessment tools to estimate complete toxicokinetic profiles in infants might broaden the scope of findings for specific periods of susceptibility.

Several studies have attempted to describe POP toxicokinetics in humans through various modeling approaches. Ayotte et al. (2003) developed a statistical model to predict levels of PCB-153 in Inuit infants. Their multivariate model that included maternal PCB-153 plasma lipid concentration, breast-feeding duration, and the sum of two skin-fold thicknesses (an index of infant adipose tissue mass) explained 72% of PCB-153 infant plasma concentration variance at 6 months postpartum. However, predictions were limited to a single compound at a specific age, and such data-based models might not be applicable to other ranges of exposure and populations because of genetic and life habit differences. Single-compartment first-order pharmacokinetic models were used by Lorber and Phillips (2002) and LaKind et al. (2000) to assess exposure to dioxin-like compounds through breast-feeding. Although these models generated toxicokinetic profiles in infants, they integrated infant body burden at birth and breast milk concentration as independent variables, an approach that would require the sampling of both media within an epidemiologic study context. Furthermore, such models do not allow estimations of POP levels in target tissues.

Physiologically based pharmacokinetic (PBPK) modeling enables the estimation of tissue and blood POP levels based on physiologic parameters, such as organ volume and blood flow, as well as compound physicochemical properties. PBPK models have been developed to evaluate infant exposure to 2,3,7,8-tetrachlorodibenzo-*p*-dioxin (TCDD) through breast-feeding (Ayotte et al. 1996; Gentry et al. 2003; Kreuzer et al. 1997). Although this is a relevant approach to assess POP tissue dosimetry in humans, PBPK frameworks to date have not allowed the integration of individual physiologic characteristics (e.g., body mass index lifetime profiles), thus preventing their use within the context of epidemiologic studies. Moreover, no attempts were made to validate these models on a large scale using longitudinal POP measurements and on multiple persistent chemicals. To generate individualized toxicokinetic profiles to be used within epidemiologic studies, we recently developed a PBPK framework to assess lifetime internal exposure to different POPs in women that is based on the physiology and reproductive history of the subjects (Verner et al. 2008). Based on this framework, the model presented herein was developed to generate individualized toxicokinetic profiles in infants exposed pre- and postnatally.

In this study, we aimed to develop a generic model to estimate infant exposure to POPs through placental transfer and breast-feeding and to validate the model using data on levels of three polychlorinated biphenyl (PCB) congeners [2,2′,4,4′,5,5′-hexachlorobiphenyl (PCB-153), 2,2′,3,4,4′,5,5′-heptachlorobiphenyl (PCB-180), and 2,2′,3,4,4′,5′-hexachlorobiphenyl (PCB-138)]; hexachlorobenzene (HCB); β-hexachlorocyclohexane (β-HCH); 1,1-dichloro-2,2-bis(*p*-chlorophenyl)ethylene (*p,p*′-DDE); and *p,p*′-DDT measured in mothers and infants in the course of an infant cohort study in the Inuit population of Nunavik (Northern Québec, Canada).

## Methods

The development of the PBPK model followed a four-step approach: model representation, parameterization, simulation, and validation. The PBPK model presented herein was based on a previous model (Verner et al. 2008) modified to consider additional information on events related to pregnancy, lactation, and infant physiology.

### Model representation

**Figure 1:**
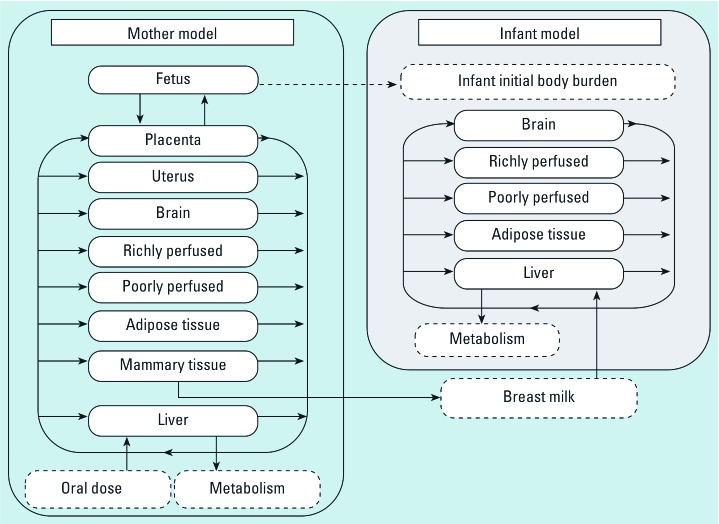
Conceptual representation of the mother–infant PBPK model. A previously published model (Verner et al. 2008) for the mother (left) was modified to integrate an infant submodel (right). Initial infant body burden was calculated as detailed in “Methods.”

The model representation for the mother was described previously (Verner et al. 2008). Briefly, the maternal model was functionally described as a tissue network of nine compartments (liver, brain, adipose tissue, richly perfused tissues, poorly perfused tissues, mammary tissue, uterus, placenta, and fetus) (Figure 1). POPs were assumed to be fully absorbed through ingestion of contaminated food and their absorption was described as a direct input into the liver compartment. Excretion through lactation was represented as output from the mammary tissue compartment.

To describe the exposure/toxicokinetics in infants, we integrated a five-compartment submodel (i.e., liver, adipose tissue, richly perfused tissue, poorly perfused tissue, brain) with the maternal model (Figure 1). Infant liver was defined as the first-pass organ where both POP intake from breast-feeding and POP metabolism take place. Adipose tissue represented the main storage compartment for POPs. Richly and poorly perfused tissue compartments represented groups of organs that were lumped according to their perfusion rates and residence times. Finally, the brain was represented as a specific compartment, because it is the target organ in relation to potential neurodevelopment deficits. The female infant model was conceptually different from the maternal model, because compartments relating to pregnancy and lactation (i.e., uterine, placental, and mammary tissues) were either physiologically irrelevant for males or kinetically irrelevant for females during the first year of life.

#### Absorption

As mentioned above, oral absorption of POPs for both mothers and infants was described as a direct input in the liver compartment. The assumption of 100% absorption is supported by high POP absorbed fractions, reported by McLachlan (1993) and Maruyama et al. (2003). We assumed dietary exposure to POPs in mothers to be a constant daily dose adjusted for body weight. Infant postnatal exposure to POPs was limited to the ingestion of contaminated breast milk up to 12 months of age. Because of the lack of information on mixed feeding (partial breast-feeding), only the exclusive breast-feeding period was considered. Infant intake was calculated as follows:


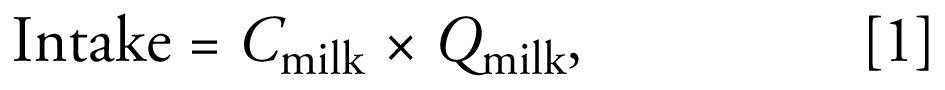


where Intake is the hourly intake through breast-feeding in micrograms per hour, *C*_milk_ is the POP concentration in milk (micrograms per liter), and *Q*_milk_ is the milk intake in liters per hour.

Infant initial body burden was based on the assumption that intrauterine exposure to POPs is blood-flow limited. Because lipophilic POPs distribution is solely driven by their solubility in lipids, infant blood and tissue lipid-adjusted concentrations at birth were equal to lipid-adjusted levels in maternal blood at the time of delivery.

#### Distribution

The distribution of POPs in compartments was managed by both the blood flow and tissue:blood partition coefficients. This process was described by a set of mass balance differential equations (MBDE) that assume homogenous distributions in tissues, as follows:


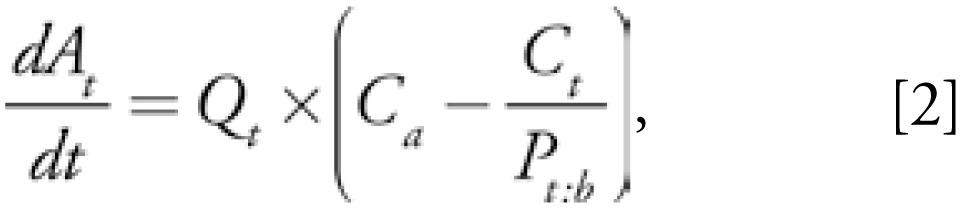


where *A_t_* represents the amount of chemical in the compartment (micrograms), *Q_t_* is the blood flow perfusing the compartment (liters per hour), *P_t:b_* is the tissue:blood partition coefficient for the compartment, and *C_a_* and *C_t_* stand for concentrations in arterial blood and the tissue (micrograms per liter), respectively. Venous and arterial blood concentrations were assumed to be equal, the latter being calculated as a weighed sum of tissues venous blood concentration:


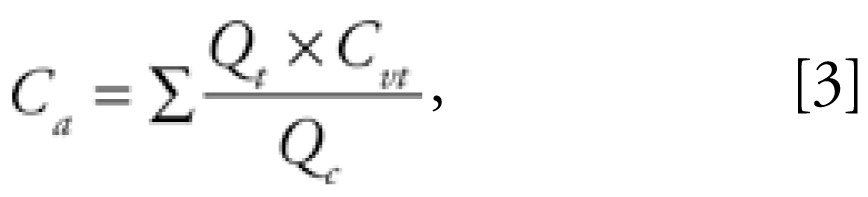


where *C_a_* is the arterial blood concentration (micrograms per liter), *Q_t_* is the blood flow to tissues (liters per hour), *C_vt_* is the tissue venous blood concentration (micrograms per liter), and *Q_c_* is the cardiac output (liters per hour).

#### Metabolism

Metabolism was limited to the liver compartment, and the rate was described by the product of the hepatic extraction ratio (*E_h_*), the liver blood flow in liters per hour (*Q_l_*), and the arterial blood concentration in micrograms per liter (*C_a_*) entering the compartment, as follows:


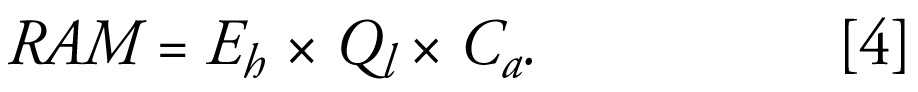


The hepatic extraction ratio was calculated as a function of liver volume adjusted intrinsic clearance in liters per hour per kilogram liver (*C_l_*_int_), blood flow to liver in liters per hour (*Q_l_*), and liver volume in liters (*V_l_* ):


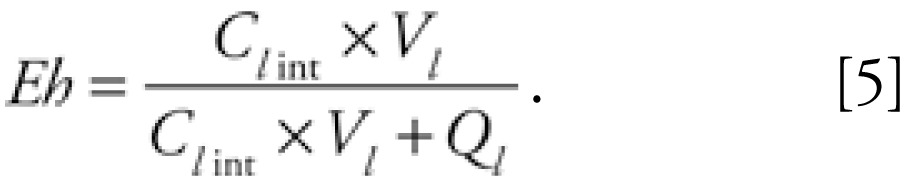


Intrinsic clearance values were assumed to be equal in mothers and infants. Ontogenic changes in enzymes catalyzing POP biotrans-formation were not included in this study but could easily be implemented in the future.

#### Excretion

Excretion of POPs through breast-feeding was detailed in our previous study (Verner et al. 2008). Briefly, POP excretion through breast milk was managed by milk flow out of the mammary tissue compartment and milk:blood partition coefficient. Placental transfer of POPs was modeled as an initial body burden in infants, where tissue and blood lipid-adjusted concentrations were equal to maternal plasma lipid-adjusted POP concentration.

### Model parameterization

#### Maternal parameters

Maternal parameters were those of the previously published model (Verner et al. 2008), with modifications for blood lipids during pregnancy as well as breast milk lipids and daily excreted volume. Blood lipid profile throughout pregnancy and postpartum period was modeled as a 70% linear increase in neutral lipids from the start of pregnancy until delivery, and a linear decrease back to normal values over 1.5 months postpartum. This profile was derived from data on triglycerides and cholesterol throughout and after pregnancy (Chiang et al. 1995).

Breast milk volume and lipid content were modified from our previously published article. A better description of ingested milk volume as a function of both infant age and infant body weight was found in Arcus-Arth et al. (2005) and was modified as follows:


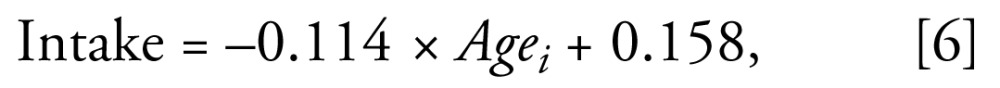


where *Age_i_* is the infant age in years and daily milk intake is in liters per kilogram of infant body weight. According to this equation, the breast milk daily intake is assumed to be 0.158 L/kg of infant body weight at birth and to decline to 0.044 L/kg of infant body weight at the age of 12 months. Breast milk lipid composition was also modified: A logarithmic function was optimized on breast milk lipid fraction data from Bitman et al. (1983) for the first 42 days and Arcus-Arth et al. (2005) for the period 3–12 months:


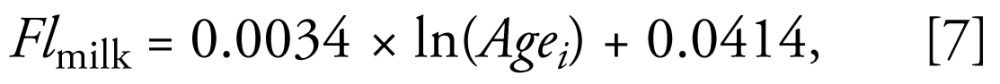


where *Fl*_milk_ represents the fraction of lipids in milk, and *Age*_i_ is the infant age in years. Given this equation, the fraction of lipids in breast milk at the time of delivery is set to approximately 0.0101 and rapidly increases to reach 0.0414 one year postpartum.

#### Infant parameters

Equations and parameters describing infant physiology were allowed to change as a function of sex, age, body weight, and body height. Age- and sex-specific organ volumes and blood flows are presented in Table 1. Tissue:blood and milk:blood partition coefficients were calculated with the approach of Haddad et al. (2000), which showed that the distribution of chemicals with log octanol:water partition coefficient (*K*_ow_) values > 4 is driven solely by lipid fractions in tissues and blood:


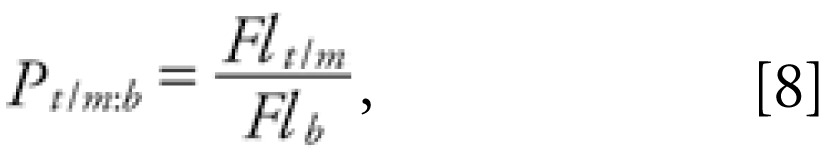


where *P_t/m:b_* is the tissue:blood or milk:blood partition coefficient, *Fl_t/m_* stands for the fraction of lipids in tissue or milk, and *Fl_b_* represents the fraction of lipids in blood. Fractions of lipids in tissues and blood are shown in Table 2. Values for richly and poorly perfused tissues were calculated from volume-adjusted lipid fractions of organs they represent. Equations and values were taken from Price et al. (2003). Because no significant variation was seen in these compartments’ lipid fractions during the first year of life, these values were kept constant.

Metabolism was parameterized from half-life values as described previously (Verner et al. 2008). Briefly, half-life values were transformed into liver volume-adjusted intrinsic clearance values (Table 3) to calculate hepatic extraction ratios.

### Model simulation and validation

#### Variability assessment

To grasp the possible range of under- and overprediction we may expect due to interindividual variability in sensitive physiologic parameters [sensitivity analysis shown in the Supplemental Material (online at http://www.ehponline.org/members/2008/0800047/suppl.pdf)], we performed Monte Carlo analyses on three independent sensitive parameters relevant to POP toxicokinetics for infants exposed through breast-feeding (100 iterations). In this analysis, published SD values for the fraction of lipids in infant adipose tissue (0.12), the fraction of lipids in breast milk (0.0032), and the daily ingested breast milk consumption (0.038 L/kg infant body weight/day) were used to randomly assign parameter values in each iteration, assuming normal distributions (Arcus-Arth et al. 2005; Bitman et al. 1983; White et al. 1991).

We used body weight and height profiles for the 50th percentile taken from the Centers for Disease Control and Prevention (CDC) data as surrogate physiologic profiles for both mother and infant (Kuczmarski et al. 2000). The analysis was done for a woman giving birth to a girl at the arbitrary age of 25, followed by a 3-month breast-feeding period. Mothers were exposed to 10 ng/kg body weight/day of the highest and lowest half-life compounds used in this study (PCB-153 and *p,p*′-DDT).

#### Validation data set

Validation of the model was done using data on PCB-180, PCB-153, PCB-138, HCB, β-HCH, *p,p*′-DDE, and *p,p*′-DDT levels in Inuit mothers and infants from northern Québec (Canada). The cohort was described previously by Muckle et al. (2001). The subjects kept for the validation step were those for which sufficient information was available:

- Mother physiology (prepregnancy weight, height, age at delivery)
- History of lactation (exclusive breastfeeding period)
- Infant physiology (body weight and body height information for a period covering the time of infant plasma sampling)
- Dates of sampling (mother and infant plasma)
- POP concentrations in mothers and infants at 6 months of age (with levels above the limit of detection).

#### Simulations

To run lifetime exposure in each mother, the PBPK model required complete body weight and body height profiles. Because maternal body height and weight were available only at one time before pregnancy, we used growth curves to infer lifetime profiles. This was done by assigning a percentile body height or body height profile from CDC data to fit the individual prepregnancy body weight and height. Maternal exposure was optimized to fit the lipid-adjusted POP level measured in plasma. Infant body weight and height were linearly interpolated between measurements taken at 0, 6, and 12 months. Simulated lipid-adjusted infant plasma (at 6 months of age), breast milk, and cord blood levels were then compared with measured levels through correlation analyses.

#### Software

We performed PBPK modeling and simulations using ACSLXtreme software (Aegis Technologies Group, Inc., Huntsville, AL, USA). We carried out nonparametric correlation analyses (Spearman coefficients) using SPSS for Windows statistical package (SPSS Inc., Chicago, IL, USA).

## Results

### Monte Carlo simulations

**Figure 2:**
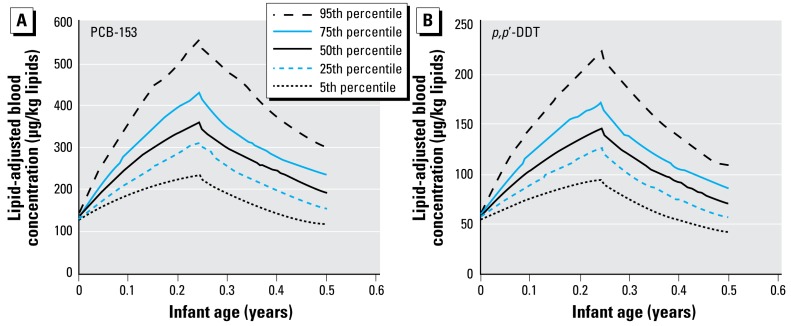
Blood POP level distributions in infants obtained from Monte Carlo simulations for (*A*) PCB-153 and (*B*) *p,p ′*-DDT. Simulations were carried out by varying three independent sensitive parameters (100 iterations). Toxicokinetic profiles were simulated for a mother exposed to a constant dose of 10 ng/kg/day and giving birth to a girl at 25 years of age, followed by a 3-month breast-feeding period. Simulations were performed for the highest and lowest half-life compounds in this study.

We assessed population variability for given physiologic profiles and breast-feeding history using Monte Carlo simulations based on variability of three independent sensitive parameters (breast milk consumption, fraction of lipids in breast milk, and fraction of lipids in infant adipose tissue). Simulations for PCB-153 and *p,p*′-DDT yielded distributions of toxicokinetic profiles in infants during their first 6 months (Figure 2). At the end of the breast-feeding period (3 months postpartum), the 5th–95th percentile values in infant blood concentration were 227–538 μg/kg lipids for PCB-153 and 90–213 μg/kg lipids for *p,p*′-DDT. The 5th–95th percentile ranges at 6 months of age were 119–303 and 42–109 μg/kg lipids for PCB153 and *p,p*′-DDT, respectively. These ranges indicated an approximately 2.5-fold variability between the 5th and 95th percentiles.

### Simulation of maternal POP toxicokinetics

To simulate the lifetime toxicokinetics of POPs in mothers, exposure was optimized to fit plasma concentration in samples drawn around the time of delivery. Lowest and highest exposure values used to match maternal levels were kept for the determination of environmental exposure ranges. Estimated maternal exposure ranges were (ng/kg body weight/day) as follows: 1.3–52.3 for PCB-153; 1.5–31.8 for PCB-180; 0.9–34.9 for PCB-138; 1.6–58.9 for HCB; 0.2–4.4 for β-HCH; 5.2–212.5 for *p,p*′-DDE; and 0.4–25.7 for *p,p*′-DDT.

### Prediction of cord blood concentration

**Figure 3:**
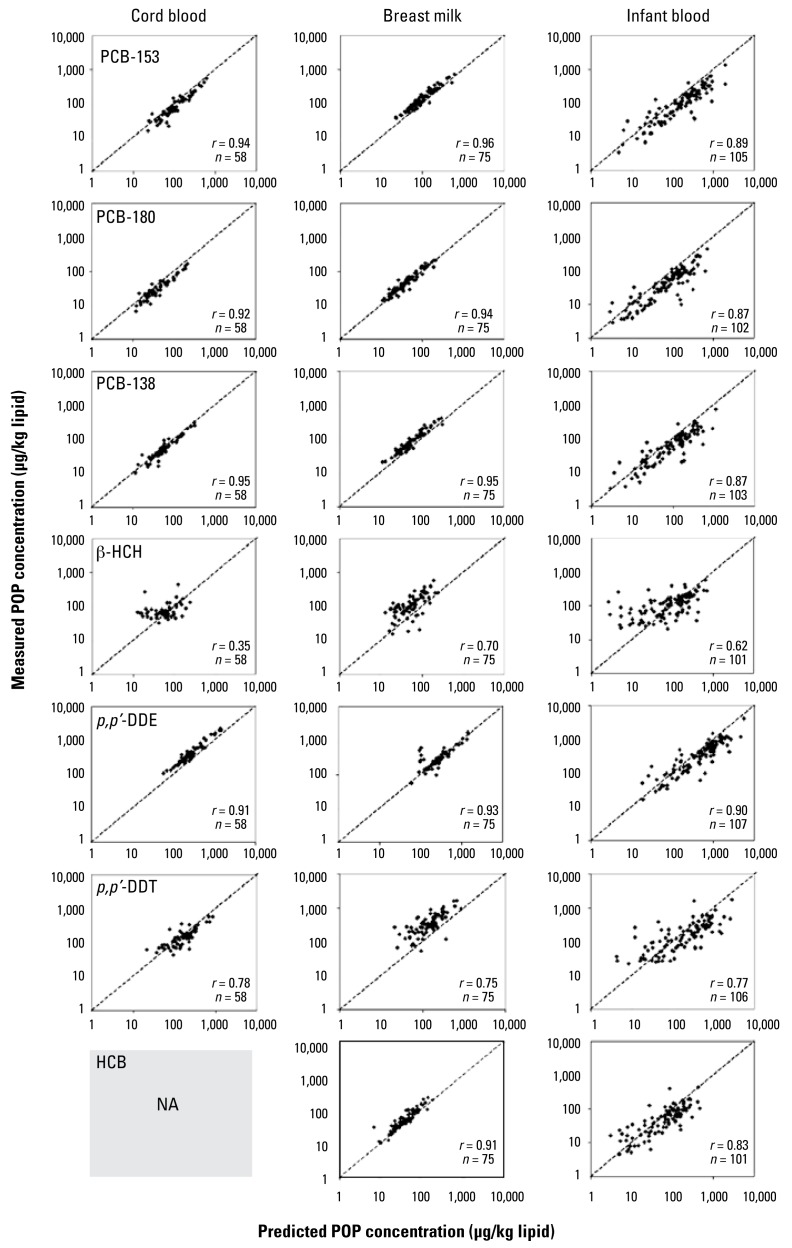
Spearman’s correlations between predicted and measured lipid adjusted POP levels in infant plasma, cord blood, and breast milk. NA, not applicable. Dotted lines represent the unity slope. Correlation analysis for HCB levels in cord blood could not be conducted because this compound was not measured in this media.

As mentioned above, cord blood lipid-adjusted concentration was assumed to be the same as maternal lipid-adjusted blood concentration at the time of delivery. Using this assumption, strong Spearman’s correlations (*r* > 0.90) were obtained for most POPs when comparing simulated values with measured values, as depicted in Figure 3. Weaker correlations were observed for *p,p*′-DDT (*r* = 0.79) and β-HCH (*r* = 0.35). No systematic under- or overestimation was observed for cord blood levels prediction. Comparison could not be achieved for HCB, as this chemical was not analyzed in cord blood.

### Prediction of breast milk concentration

Because breast-feeding was considered to be the exclusive route of postnatal exposure in infants for this study, simulated breast milk concentrations were compared with levels in samples collected approximately 1 month after delivery. Most correlations had Spearman’s rho values above 0.90 (Figure 3). *p,p*′-DDT and β-HCH simulated breast milk levels had weaker correlation values of 0.72 and 0.70, respectively. A slight tendency to underestimate breast milk concentrations was observed for most compounds.

### Estimation of infant blood concentration at ~ 6 months of age

Infant blood levels were predicted considering both exposure through placental transfer and breast-feeding. Simulated toxicokinetic profiles matched measured infant blood POP concentration with high Spearman’s correlations (Figure 3). Plots of measured values on predicted values showed a slight systematic overestimation (Figure 3). Simulations of β-HCH toxicokinetics in infants yielded inconsistent results, displaying a high variability that could not be explained by the model at low concentrations. Predicted blood concentrations fell within the 2.5-fold range variation defined in Monte Carlo simulations (between the 5th and 95th percentiles) for 88% (*p,p*′-DDE), 85% (PCB-153 and PCB-138), 82% (HCB), 75% (PCB-180), 67% (*p,p*′-DDT), and 66% (β-HCH) of individuals.

## Discussion

In this study we aimed to build and validate a PBPK model for the characterization of infant exposure to POPs through placental transfer and breast-feeding. This work follows our first modeling paper on the assessment of lifetime POP toxicokinetics in human (Verner et al. 2008) and focuses primarily on exposure in infants. The successful validation of our PBPK model within this study using data on mothers and infants from an Inuit population further supports the potential of this internal exposure assessment tool in epidemiologic studies.

**Figure 4:**
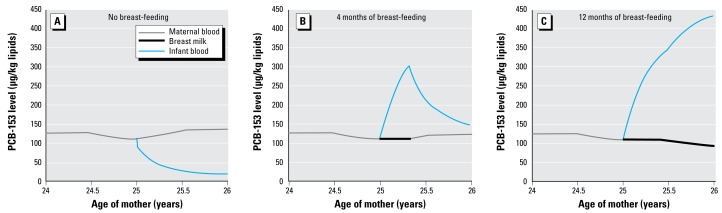
Examples of toxicokinetic profiles for PCB-153 in maternal blood, breast milk, and infant blood for different breast-feeding scenarios.

This PBPK model based on individual physiology and breast-feeding period allows the integration of several concurrent physiologic events (e.g., pre- and postpartum changes in maternal physiology, lactation, infant growth) that are relevant to POP toxicokinetics. Modeling simultaneous variations in volume and lipid composition of maternal tissues and breast milk over time is critical in characterizing POP distribution and thus infant exposure. The determination of infant initial body burden is also essential to correctly assess perinatal exposure, because initial levels rapidly depurate due to infant growth. The toxicokinetic profiles depicted in Figure 4 show the close relationship between POP levels in maternal blood, breast milk, and infant blood. Another benefit of using PBPK modeling is the possibility of predicting POP concentration in potential target tissues of newborns and infants. However, such estimates were not shown in the present study, as their validation would have required the sampling of infant tissues.

To estimate background exposure to POPs in mothers, the daily intake was optimized so the simulated lipid-adjusted blood concentration reached the measured maternal blood level. A study by Dewailly et al. (1996) estimated the mean daily PCB exposure in Inuits to be 13.8 μg/day (i.e., a 230-ng/kg body weight/day intake for an average 60-kg woman). In their review, Van Oostdam et al. (2005) reported median daily intakes of 20 ng/kg body weight/day for HCB, 40 ng/kg body weight/day for *p,p*′-DDT, and 50 ng/kg BW/day for PCBs in Inuits of Qikiqtarjuaq. The estimated daily exposure ranges (nanograms per kilogram body weight per day) obtained in this study for *p,p*′-DDT (0.4–25.7), HCB (1.6–58.9), and the sum of the three PCBs (3.7–119) were comparable to these daily intake estimates, indicating that the PBPK model is fairly accurate at estimating the maternal exposure to POPs. As reported previously (Verner et al. 2008), daily intakes can be better assessed when considering pregnancies and lactation periods that precede the time of sampling, as well as dietary habits and temporal trends in environmental levels. When available, such information could easily be integrated in the PBPK model.

Predicted cord blood, breast milk, and infant blood concentrations by PBPK modeling showed strong Spearman’s correlations with measured levels (see Figure 3). However, a minor discrepancy still remains between simulated and measured levels, indicating that sources of toxicokinetic variability are not accounted for within the model. Monte Carlo simulations showed that the infant toxicokinetic profile is influenced by the variability in breast milk lipids and volume, as well as by the lipid fraction in the adipose tissue compartment. Variability within these sensitive parameters is not taken into account when simulating POP toxicokinetics, leading to potential errors in predictions. Simulated values fell within the 2.5-fold variability range for 66–88% of individuals used in this study, depending on the compound. This suggests that other factors such as inaccuracy in information from questionnaires and variability in the analytic methods used to quantify POPs might affect model predictability. It is also possible that some parameters were incorrectly estimated, as the physiologic equations were derived from data on Caucasians, whereas the data set used in this study was on Inuit people. The under- or overestimation of infant adipose tissue volume might yield a systematic bias and other measurements to estimate this value, such as the sum of skin folds (Ayotte et al. 2003), should be evaluated in future studies.

Another source of error can arise from the fact that exposure to POPs through the ingestion of contaminated breast milk was limited to the exclusive breast-feeding period. Because of a lack of information, the period of mixed feeding (when the infant is fed both breast milk and formula) was assumed to be a period without breast-feeding, leading to a potential underestimation of infant exposure in cases with important mixed feeding. A detailed description of formula and milk consumption history would provide valuable information for the estimation of exposure through breast-feeding during the mixed feeding period and allow the inclusion of partial breast-feeding in the model.

Correlations between predicted and measured levels were weaker for β-HCH and *p,p*′-DDT. These compounds are characterized by their shorter half-lives and levels in mothers and infants that are near the limits of detection. The impact of half-life alone on model inability to precisely estimate measured values is not likely to be the most influent factor, given that simulated levels of HCB (a compound with a shorter half-life than β-HCH) were strongly correlated to measured concentrations in infant plasma and breast milk. On the other hand, model accuracy might be limited when working with POP levels close to limits of detection, a phenomenon potentially caused by reduced precision in analytic methods at low POP concentration in samples. Predictions with β-HCH might have been influenced by the fact that this compound has a lower log *K*_ow_ (3.81). Its value below the partitioning cutoff (i.e., log *K*_ow_ = 4) for the method used in this study possibly led to a slight error in partition coefficients determination. Overall, caution should be exerted when using this model for compounds with levels near the limits of detection and/or that have low log *K*_ow_ values.

Simulations slightly underestimated POP lipid-adjusted concentration in breast milk. This might be explained by the difference in approaches to adjust POP levels for lipids. Adjustment for blood lipids in the Ayotte et al. (2003) study was based on total lipids as described in Phillips et al. (1989), whereas the PBPK model used neutral lipid equivalents exclusively. Lipids in breast milk are composed almost solely of triacylglycerols (Jensen 1999), whereas lipids in maternal blood also contain significant levels of cholesterol and phospholipids (Berghaus et al. 1998). Although triacylglycerols and cholesterol are neutral lipids in which POP will be stored, phospholipids have a lipohydrophilicity similar to a mixture of 30% neutral lipids and 70% water (Poulin and Krishnan 1995). Therefore, including phospholipids in blood lipid content calculation leads to an artifactual difference between blood and milk lipid-adjusted POP concentrations. As only neutral lipid equivalents are considered in the PBPK model and POPs are assumed to be homogeneously distributed in lipids, simulated lipid-adjusted POP levels in blood and milk are equal. Thus, the underestimation of POP levels in breast milk can be explained at least partially by these different approaches in adjusting levels for lipid contents. When information on whole blood and breast milk lipid composition is available, levels should be adjusted on neutral lipid equivalents rather than total lipids.

**Figure 5:**
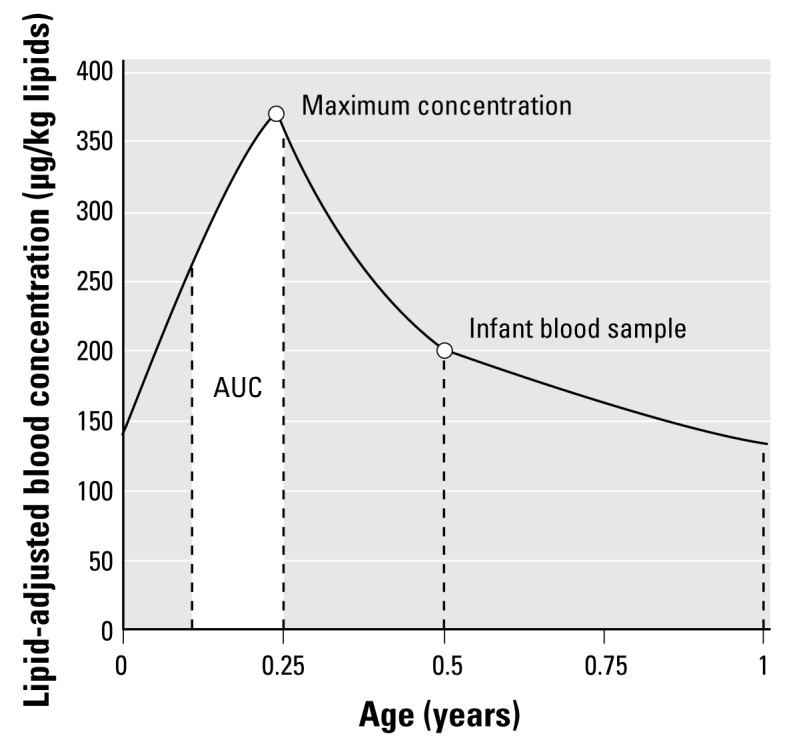
Graphic representation of information to be harvested from simulations. The maximum concentration and the area under the curve (AUC) represented by the white area are examples of information to be extracted from the toxicokinetic curve.

The results of this study showed the potential of PBPK modeling in estimating POP toxicokinetics in infants. Detailed information on internal exposure, such as the timing and amplitude of the maximum concentration, can be harvested from the simulations (Figure 5). This could be an important input in epidemiology to study high exposure during hypothesized critical time windows. For example, it was suggested that internal exposure to PCB alters thyroid hormones levels (Chevrier et al. 2007). Disturbed thyroid levels in neonates can result in several adverse health effects such as visual, motor, language, and memory impairment (Zoeller and Rovet 2004). Using only measured POP levels in 6-month-old infants to test an exposure-effect hypothesis might be too limiting for the complete analysis of time- and dose-related responses. An approach using the maximum concentration (and the time when this concentration is reached) as well as the area under the curve for different time frames might provide crucial information on critical windows of exposure as well as dose–response relationships.

Overall, PBPK modeling was shown to be a relevant method to assess pre- and postnatal exposure to POPs. The presented model allows the prediction of infant exposure through placental transfer (cord blood level estimation at the time of delivery) and breast-feeding strictly from information on maternal blood levels and physiologic profiles that can be easily gathered from epidemiologic questionnaires. Moreover, this is the first study to validate a PBPK model of POPs in infants on an individual basis. This study also successfully demonstrates how our previously published model (Verner et al. 2008) adequately describes the lactational excretion of POPs in women. This PBPK modeling approach will permit the assessment of exposure to POPs in infants prospectively and retrospectively, therefore reducing sampling efforts and enabling the use of individualized POP toxicokinetic profiles in epidemiologic studies. Further research is planned to validate the model with other populations, later life stages, and additional compounds such as PBDEs. Researchers interested in collaborating with us or using our model are encouraged to contact us.

## Figures and Tables

**Table 1:** Physiologic parameters for male and female infants from birth to 12 months of age.

Parameters	Sex	Equations
BS^a^	M & F	BW^0.515^ × (BH^0.422^) × 234.9
Volumes (L)		
Liver (Vl)^a^	M & F	0.05012 × BW^0.780^
Adipose tissue (Vat)^a^	M & F	0.91 × BW − (Vl + Vrp + Vpp + Vsk)
Skin tissue (Vsk)^a^	M & F	0.664 × (BS/10^4^) + 0.07850 × (BS/10^4^)^1.049^
Richly perfused tissue (Vrp)^a^	M	−0.01068 × AGE_i_ + 2.038 × (BW^2^/BH)^0.4014^ − 0.2046 −Vl
	F	−0.01919 × AGE_i_ + 3.193 × (BW^2^/BH)^0.2657^ − 1.374 −Vl
Poorly perfused tissue (Vpp)^a^	M & F	Vt + Vh + Vsm
Tongue (Vt)^a^	M & F	0.00119 × BW −0.0004302
Heart (Vh)^a^	M	0.0000001017 × [(BH^0.6640^) × (BW^0.3851^) × 242.7]^1.420^
	F	0.0000001017 × [(BH^0.6862^) × (BW^0.3561^) × 242.7]^1.420^
Skeletal muscle (Vsm)^a^	M	0.09561 × BW + 0.01601 × BH + 0.1097 × AGE_i_
	F	0.09563 × BW + 0.01650 × BH + 0.09102 × AGE_i_ − 0.1642
Brain (Vbrain)^b^	M	10 × (AGE_i_ + 0.213)/(6.030 + 6.895 × AGE_i_)
	F	10 × (AGE_i_ + 0.226)/(6.521 + 7.514 × AGE_i_)
Blood flows (L/hr)		
Cardiac output (Qc)^a^	M	0.2519 × BW^0.7609^ × 60
	F	0.2508 × BW^0.7815^ × 60
Liver (Ql)^a^	M	0.84 × Vl × 60
	F	Vl × 60
Adipose tissue (Qat)^a^	M	0.0209 × Vat × 60
	F	0.0300 × Vat × 60
Richly perfused (Qrp)^a^	M & F	QC − (Qpp + Qat + Ql + Qbrain)
Poorly perfused (Qpp)^a^	M	(0.03 × (Vt + Vsm) + 0.73 × Vh) × 60
	F	(0.03 × (Vt + Vsm) + 0.96 × Vh) × 60
Brain (Qbrain)^c^	M & F	− 0.0024 × AGE_i_4 + 0.1305 × AGE_i_3 − 2.4822 × AGE_i_2 + 18.025 × AGE_i_ + 15.197

Abbreviations: AGEi, age; BH, body height; BS, body surface; BW, body weight; F, female; M, male; Qat, blood flow to adipose tissue; Qbrain, blood flow to brain; Qc, cardiac output; Ql, blood flow to liver; Qpp, blood flow to poorly perfused tissue; Qrp, blood flow to richly perfused tissue; Vat, adipose tissue volume; Vbrain, brain volume; Vh, heart volume; Vl, liver volume; Vpp, poorly perfused tissue volume; Vrp, richly perfused tissue volume; Vsk, skin volume; Vsm, skeletal muscle volume; Vt, tongue volume. Equations are based on infant body weight, body height, body surface, and age.

a Modified from Haddad et al. (2006).

b Modified from Haddad et al. (2001).

c Data from Price et al. (2003)

**Table 2:** Fraction of lipids in infant tissues.

Tissue	Age (years)	Fraction of lipids
Blood^a^	0–1	0.005
Adipose tissue^a^	0	0.347
	0.5	0.472
	9	0.550
Liver^a^	0	0.021
	1	0.041
Richly perfused^b^	0–1	0.018
Poorly perfused^b^	0–1	0.021
Brain^a^	0	0.026
	1.5	0.061

a Data from White et al. (1991).

b Calculated as detailed in “Methods.”

**Table 3:** Half-life and calculated liver volume–adjusted intrinsic clearance of POPs in infants and mothers.

Compounds	Half-life (years)	Intrinsic clearance (L/hr/kg liver)	Source of half-life value
PCB-153	27.5	0.0082601	Yakushiji et al. (1984)
PCB-180	9.9	0.0229504	Yakushiji et al. (1984)
PCB-138	16.3	0.0144328	Yakushiji et al. (1984)
HCB	6	0.0395820	To-Figueras et al. (1997)
β-HCH	7.6	0.0309631	Jung et al. (1997)
*p,p*′-DDE	15	0.0156840	Wolff et al. (2000)
*p,p*′-DDT^a^	5	0.0144328	Smith (1999)

a The half-life value displayed was chosen arbitrarily for a range of 4.2–5.6 years in Smith (1999).

## References

[b1-ehp-117-481] Arcus-Arth A, Krowech G, Zeise L (2005). Breast milk and lipid intake distributions for assessing cumulative exposure and risk. J Expo Anal Environ Epidemiol.

[b2-ehp-117-481] Ayotte P, Carrier G, Dewailly É (1996). Health risk assessment for Inuit newborns exposed to dioxin-like compounds through breast feeding. Chemosphere.

[b3-ehp-117-481] Ayotte P, Muckle G, Jacobson JL, Jacobson SW, Dewailly É (2003). Assessment of pre- and postnatal exposure to polychlorinated biphenyls: lessons from the Inuit Cohort Study. Environ Health Perspect.

[b4-ehp-117-481] Berghaus TM, Demmelmair H, Koletzko B (1998). Fatty acid composition of lipid classes in maternal and cord plasma at birth. Eur J Pediatr.

[b5-ehp-117-481] Bitman J, Wood L, Hamosh M, Hamosh P, Mehta NR (1983). Comparison of the lipid composition of breast milk from mothers of term and preterm infants. Am J Clin Nutr.

[b6-ehp-117-481] Chevrier J, Eskenazi B, Bradman A, Fenster L, Barr DB (2007). Associations between prenatal exposure to polychlorinated biphenyls and neonatal thyroid-stimulating hormone levels in a Mexican-American population, Salinas Valley, California. Environ Health Perspect.

[b7-ehp-117-481] Chiang AN, Yang ML, Hung JH, Chou P, Shyn SK, Ng HT (1995). Alterations of serum lipid levels and their biological relevances during and after pregnancy. Life Sci.

[b8-ehp-117-481] Dewailly É, Ayotte P, Blanchet C, Grondin J, Bruneau S, Holub B (1996). Weighing contaminant risks and nutrient benefits of country food in Nunavik. Arctic Med Res.

[b9-ehp-117-481] Eskenazi B, Rosas LG, Marks AR, Bradman A, Harley K, Holland N (2008). Pesticide toxicity and the developing brain. Basic Clin Pharmacol Toxicol.

[b10-ehp-117-481] Gentry RP, Covington TR, Clewell HJ (2003). Evaluation of the potential impact of pharmacokinetic differences on tissue dosimetry in offspring during pregnancy and lactation. Regul Toxicol Pharm.

[b11-ehp-117-481] Haddad S, Poulin P, Krishnan K (2000). Relative lipid content as the sole mechanistic determinant of the adipose tissue:blood partition coefficients of highly lipophilic organic chemicals. Chemosphere.

[b12-ehp-117-481] Haddad S, Restieri C, Krishnan K (2001). Characterization of age-related changes in body weight and organ weights from birth to adolescence in humans. J Toxicol Environ Health A.

[b13-ehp-117-481] Haddad S, Tardif GC, Tardif R (2006). Development of physiologically based toxicokinetic models for improving the human indoor exposure assessment to water contaminants: trichloroethylene and trihalomethanes. J Toxicol Environ Health A.

[b14-ehp-117-481] Jensen RG (1999). Lipids in human milk. Lipids.

[b15-ehp-117-481] Jung D, Becher H, Edler L, Flesch-Janys D, Gurn P, Konietzko J (1997). Elimination of beta-hexachlorocyclohexane in occupationally exposed persons. J Toxicol Environ Health.

[b16-ehp-117-481] Koopman-Esseboom C, Weisglas-Kuperus N, de Ridder MA, Van der Paauw CG, Tuinstra LG, Sauer PJ (1996). Effects of polychlorinated biphenyl/dioxin exposure and feeding type on infants’ mental and psychomotor development. Pediatrics.

[b17-ehp-117-481] Kreuzer PE, Csanady GA, Baur C, Kessler W, Papke O, Greim H (1997). 2,3,7,8-Tetrachlorodibenzo-*p*-dioxin (TCDD) and congeners in infants. A toxicokinetic model of human lifetime body burden by TCDD with special emphasis on its uptake by nutrition. Arch Toxicol.

[b18-ehp-117-481] Kuczmarski RJ, Ogden CL, Grummer-Strawn LM, Flegal KM, Guo SS, Wei R (2000). CDC Growth Charts: United States Advance Data from Vital and Health Statistics No 314.

[b19-ehp-117-481] LaKind JS, Berlin CM, Park CN, Naiman DQ, Gudka NJ (2000). Methodology for characterizing distributions of incremental body burdens of 2,3,7,8-TCDD and DDE from breast milk in North American nursing infants. J Toxicol Environ Health A.

[b20-ehp-117-481] Lorber M, Phillips L (2002). Infant exposure to dioxin-like compounds in breast milk. Environ Health Perspect.

[b21-ehp-117-481] Maruyama W, Yoshida K, Tanaka T, Nakanishi J (2003). Simulation of dioxin accumulation in human tissues and analysis of reproductive risk. Chemosphere.

[b22-ehp-117-481] McLachlan MS (1993). Digestive tract absorption of polychlorinated dibenzo-*p*-dioxins, dibenzofurans, and biphenyls in a nursing infant. Toxicol Appl Pharmacol.

[b23-ehp-117-481] Muckle G, Ayotte P, Dewailly É, Jacobson SW, Jacobson JL (2001). Determinants of polychlorinated biphenyls and methylmercury exposure in Inuit women of childbearing age. Environ Health Perspect.

[b24-ehp-117-481] Phillips DL, Pirkle JL, Burse VW, Bernert JT, Henderson LO, Needham LL (1989). Chlorinated hydrocarbon levels in human serum: effects of fasting and feeding. Arch Environ Contam Toxicol.

[b25-ehp-117-481] Poulin P, Krishnan K (1995). A biologically-based algorithm for predicting human tissue: blood partition coefficients of organic chemicals. Hum Exp Toxicol.

[b26-ehp-117-481] Price K, Haddad S, Krishnan K (2003). Physiological modeling of age-specific changes in the pharmacokinetics of organic chemicals in children. J Toxicol Environ Health A.

[b27-ehp-117-481] Ribas-Fito N, Sala M, Kogevinas M, Sunyer J (2001). Polychlorinated biphenyls (PCBs) and neurological development in children: a systematic review. J Epidemiol Community Health.

[b28-ehp-117-481] Rosas LG, Eskenazi B (2008). Pesticides and child neurodevelopment. Curr Opin Pediatr.

[b29-ehp-117-481] Smith D (1999). Worldwide trends in DDT levels in human breast milk. Int J Epidemiol.

[b30-ehp-117-481] To-Figueras J, Sala M, Otero R, Barrot C, Santiago-Silva M, Rodamilans M (1997). Metabolism of hexachlorobenzene in humans: association between serum levels and urinary metabolites in a highly exposed population. Environ Health Perspect.

[b31-ehp-117-481] Van Oostdam J, Donaldson SG, Feeley M, Arnold D, Ayotte P, Bondy G (2005). Human health implications of environmental contaminants in Arctic Canada: a review. Sci Total Environ.

[b32-ehp-117-481] Verner MA, Charbonneau M, López-Carrillo L, Haddad S (2008). Physiologically-based pharmacokinetic modeling of persistent organic pollutants for lifetime exposure assessment: a new tool in breast cancer epidemiologic studies. Environ Health Perspect.

[b33-ehp-117-481] Walkowiak J, Wiener JA, Fastabend A, Heinzow B, Kramer U, Schmidt E (2001). Environmental exposure to polychlorinated biphenyls and quality of the home environment: effects on psychodevelopment in early childhood. Lancet.

[b34-ehp-117-481] White DR, Widdowson EM, Woodard HQ, Dickerson JW (1991). The composition of body tissues (II). Fetus to young adult. Br J Radiol.

[b35-ehp-117-481] Wolff MS, Zeleniuch-Jacquotte A, Dubin N, Toniolo P (2000). Risk of breast cancer and organochlorine exposure. Cancer Epidemiol Biomarkers Prev.

[b36-ehp-117-481] Yakushiji T, Watanabe I, Kuwabara K, Tanaka R, Kashimoto T, Kunita N (1984). Rate of decrease and half-life of polychlorinated biphenyls (PCBs) in the blood of mothers and their children occupationally exposed to PCBs. Arch Environ Contam Toxicol.

[b37-ehp-117-481] Zoeller RT, Rovet J (2004). Timing of thyroid hormone action in the developing brain: clinical observations and experimental findings. J Neuroendocrinol.

