# Erratum: “A Physiologically Based Pharmacokinetic Model for the Assessment of Infant Exposure to Persistent Organic Pollutants in Epidemiologic Studies”

**DOI:** 10.1289/ehp.121-a209

**Published:** 2013-07-01

Verner et al. have reported an error in their article “A Physiologically Based Pharmacokinetic Model for the Assessment of Infant Exposure to Persistent Organic Pollutants in Epidemiologic Studies ” [Environ Health Perspect 117:481–487 (2009)]. In Figure 1, the arrows on the right pointing toward the placenta (mother model) and brain (infant model) should have pointed away from the placenta and brain. The corrected figure appears below and has also been corrected online.

**Figure:**
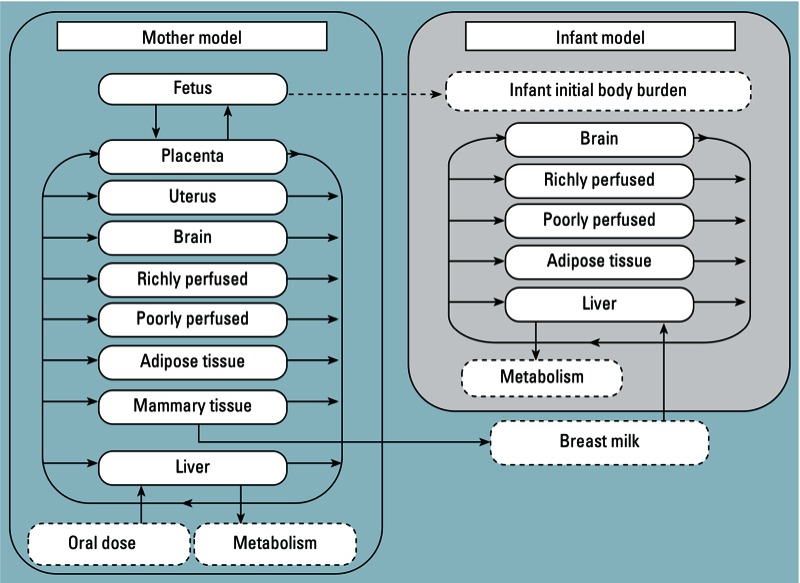
Conceptual representation of the mother–infant PBPK model. A previously published model (Verner et al. 2008) for the mother (left) was modified to integrate an infant submodel (right). Initial infant body burden was calculated as detailed in “Methods.”

*EHP* regrets the error.

